# Randomized trial of azithromycin to eradicate *Ureaplasma* respiratory colonization in preterm infants: 2-year outcomes

**DOI:** 10.1038/s41390-021-01437-2

**Published:** 2021-03-03

**Authors:** Rose M. Viscardi, Michael L. Terrin, Laurence S. Magder, Natalie L. Davis, Susan J. Dulkerian, Ken B. Waites, Marilee Allen, Ajoke Ajayi-Akintade, Namasivayam Ambalavanan, David A. Kaufman, Pamela Donohue, Deborah J. Tuttle, Jörn-Hendrik Weitkamp

**Affiliations:** 1 Department of Pediatrics, University of Maryland, Baltimore School of Medicine, Baltimore, MD USA; 2 Department of Epidemiology and Preventive Medicine, University of Maryland, Baltimore School of Medicine, Baltimore, MD USA; 3 Departments of Pathology and Pediatrics, University of Alabama at Birmingham School of Medicine, Birmingham, AL USA; 4 Department of Pediatrics, Johns Hopkins University School of Medicine, Baltimore, MD USA; 5 Mount Washington Pediatric Hospital, Baltimore, MD USA; 6 Department of Pediatrics, University of Virginia School of Medicine, Charlottesville, VA USA; 7 Department of Pediatrics, Christiana Care Health System, Newark, DE USA; 8 Department of Pediatrics, Vanderbilt University Medical Center, Nashville, TN USA

## Abstract

**Background:**

To assess the potential impact of azithromycin treatment in the first week following birth on 2-year outcomes in preterm infants with and without *Ureaplasma* respiratory colonization who participated in a double-blind, placebo-controlled randomized controlled trial.

**Methods:**

Respiratory morbidity was assessed at NICU discharge and at 6, 12, and 22–26 months corrected age using pulmonary questionnaires. Comprehensive neurodevelopmental assessments were completed between 22 and 26 months corrected age. The primary and secondary composite outcomes were death or severe respiratory morbidity and death or moderate–severe neurodevelopmental impairment, respectively, at 22–26 months corrected age.

**Results:**

One hundred and twenty-one randomized participants (azithromycin, *N* = 60; placebo, *N* = 61) were included in the intent-to-treat analysis. There were no significant differences in death or serious respiratory morbidity (34.8 vs 30.4%, *p* = 0.67) or death or moderate–severe neurodevelopmental impairment (47 vs 33%, *p* = 0.11) between the azithromycin and placebo groups. Among all trial participants, tracheal aspirate *Ureaplasma*-positive infants experienced a higher frequency of death or serious respiratory morbidity at 22–26 months corrected age (58%) than tracheal aspirate *Ureaplasma*-negative infants (34%) or non-intubated infants (21%) (*p* = 0.028).

**Conclusions:**

We did not observe strong evidence of a difference in long-term pulmonary and neurodevelopment outcomes in preterm infants treated with azithromycin in the first week of life compared to placebo.

**Impact:**

- No strong evidence of a difference in long-term pulmonary and neurodevelopment outcomes was identified at 22–26 months corrected age in infants treated with azithromycin in the first week of life compared to placebo.
- The RCT is the first study of 2-year pulmonary and neurodevelopmental outcomes of azithromycin treatment in ELGANs.
- Provides evidence that ELGANs with lower respiratory tract *Ureaplasma* have the most frequent serious respiratory morbidity in the first 2 years of life, suggesting that a Phase III trial of azithromycin to prevent BPD targeting this population is warranted.

## Introduction

Infants with bronchopulmonary dysplasia (BPD) are at risk for adverse pulmonary outcomes during childhood and into adulthood.^1–3^ Up to 50% of BPD infants require re-hospitalization in the first year of life.^4–6^ Since respiratory health may continue to evolve over the first few years of life and BPD and adverse neurodevelopmental outcomes are closely linked,^7,8^ it is essential to include long-term pulmonary and neurodevelopmental assessments as part of any neonatal clinical trial to prevent BPD.

The genital mollicute species *Ureaplasma parvum* and *Ureaplasma urealyticum* are associated with adverse pregnancy outcomes^9^ and morbidities of prematurity, including BPD,^10^ necrotizing enterocolitis,^11^ and severe intraventricular hemorrhage (IVH).^12–14^ Although macrolides have antimicrobial and immmumodulatory properties making them ideal therapeutic candidates for *Ureaplasma* eradication, none of the prior studies of macrolides for prevention of BPD included long-term follow-up.^15–17^

We conducted a series of pharmacokinetic/pharmacodynamic open-label studies of intravenous azithromycin (AZM)^18–20^ to select a safe, effective dose for the recently completed pilot (Phase IIb) randomized clinical trial of multi-dose AZM (20 mg/kg × 3 days) in extremely low gestational age newborns (ELGANs, 24–28 weeks gestation).^21^ The results of this trial demonstrated that (1) a 3-day course of AZM effectively eradicated *Ureaplasma* in ELGAN infants and (2) perinatal mortality and prolonged respiratory support are concentrated in ELGANs who have *Ureaplasma* in the lower respiratory tract. To determine the long-term safety of neonatal AZM therapy and the potential impact on long-term outcomes, pulmonary and neurodevelopmental outcomes were assessed at 22–26 months corrected age in participants of the randomized controlled trial (RCT).

## Methods

### Study design

The study design was a prospective, randomized, double-blind, placebo-controlled trial (clinicaltrials.gov NCT01778634) as recently described.^21^ The sample size for the trial was calculated to provide good power to detect moderate differences in the primary outcome of *Ureaplasma*-free survival to discharge. The U.S. Food and Drug Administration (IND78990) and the Institutional Review Board of each participating institution approved the study protocol including follow-up. Written parental consent was obtained for all participants prior to randomization. One hundred and twenty-one ELGAN infants (gestational age 24^0^ to 28^6^ weeks) were enrolled and randomized at 7 U.S. centers between July 2013 and August 2016.^21^ After baseline respiratory samples were obtained, participants were randomized to receive either intravenous AZM (American Pharmaceuticals Partners, Schaumburg, IL) 20 mg/kg at a concentration of 2 mg/ml in 5% dextrose water or equal volume of 5% dextrose water (10 ml/kg) as a placebo administered every 24 h for 3 doses. Randomization was performed using permuted block design with stratification by site and gestational age (24^0^–26^6^ vs 27^0^–28^6^ weeks) and assigned in 1:1 ratio to AZM or placebo with twins assigned to the same treatment. Since rapid diagnostic testing for *Ureaplasma* spp. was not feasible, presence of *Ureaplasma* colonization was unknown prior to dosing. All tracheal and nasopharyngeal aspirate samples were frozen at −80 °C at each study site and batch-shipped to the University of Alabama at Birmingham Diagnostic Mycoplasma Laboratory for *Ureaplasma* culture, species-specific real-time PCR and AZM susceptibility testing. *Ureaplasma* eradication was defined as three negative cultures post-treatment.^21^

### Assessments

Data collected at baseline and during hospitalization included demographic variables such as gestational age, birth weight, sex, race, and ethnicity, and neonatal morbidities, including IVH grade, periventricular leukomalacia, necrotizing enterocolitis stage, late-onset sepsis, patent ductus arteriosus, retinopathy of prematurity stage, and postnatal steroid use, tracheostomy placement, postmenstrual age (PMA) at the time of discharge to home, and modes and duration of respiratory support.^21^ BPD was classified as physiologic based on a room air challenge,^22^ modified Shennan based on supplemental oxygen use at 36 weeks PMA or time of transfer prior to 36 weeks PMA,^23^ and NIH BPD severity definitions.^24^ Socioeconomic factors, including insurance status, maternal education and marital status, and primary language spoken at home were collected at the 22–26-month corrected age visit. For all neonatal intensive care unit (NICU) and follow-up assessments, the examiners were masked to treatment assignment and *Ureaplasma* colonization status. Only the pharmacists at each site were unblinded to treatment assignment.

### Pulmonary questionnaires

Structured parental interviews were conducted before discharge and by follow-up phone interviews at 6, 12, and 22–26 months corrected age using the validated Tucson Children’s Respiratory Study questionnaires.^25–27^ Follow-up interview windows were 5–9 months corrected age with an ideal interview date at 6 months corrected age, 10–18 months with an ideal interview date at 12 months, and 19–30 months with the ideal date at 24 months corrected age. At the time of each parental interview, the parent/caregiver was asked to provide responses based on the interval since the last interview. Information was elicited on family history of asthma and atopy, the home environment such as presence of pets and tobacco smoke exposure, the frequency and characteristics of wheezing and cough, use of respiratory medications including diuretics, nebulized bronchodilators, inhaled or systemic steroids, supplemental oxygen, and respiratory illness-related hospitalizations, physician visits, or emergency room (ER) visits. Parental report of their child experiencing cough without a cold “most of the time” during an interval was classified as chronic cough and parental report of wheezing “almost every day” during an interval was classified as chronic wheezing.

### Neurodevelopmental assessments

Surviving infants completed comprehensive neurodevelopmental assessments between 22 and 26 months corrected age. Neurologic examinations^28^ that included a standardized assessment of reflexes, muscle tone, and strength were classified as normal, suspect (mildly abnormal neurologic exam without functional impairment), abnormal with functional impairment due to noncerebral palsy, or abnormal due to cerebral palsy (CP). Gross motor performance was evaluated using the modified Gross Motor Function Classification System (GMFCS) with scores ranging from 0 (normal) to 5 (most impaired).^29^ CP severity was classified as mild (GMFCS level 1), moderate (GMFSC levels 2–3), or severe (GMFCS levels 4–5).^30^ Certified examiners assessed cognitive function using the Bayley Scales of Infant and Toddler Development, third edition (BSID-III).^31^ For participants whose parents were unable to bring them to the clinic for in-person assessments, a parental phone interview was conducted to complete the Ages and Stages Questionnaire, Third Edition (ASQ3) to score developmental milestones in five domains (communication, fine motor, gross motor, problem-solving ability, and personal–social functioning).^32^ Hearing impairment (inability to understand the examiner’s verbal directions and to communicate, with or without amplification) were assessed by observation, parental report, or follow-up audiologic assessments.^33^ Vision impairment defined as vision worse than 20/200 was obtained by parental report or post-discharge eye exams. If unable to complete either in-person assessments or the phone interview ASQ3, information was extracted from chart review to classify the child for neurodevelopmental impairment (NDI).

### Outcomes

The primary outcome for the follow-up phase of the trial was the composite outcome of death prior to 26 months corrected age or serious respiratory morbidity, defined as the occurrence of one or more of the following: tracheostomy; continued hospitalization for respiratory reasons at or beyond 50 weeks PMA, use of supplemental oxygen or respiratory support at 22–26 months corrected age, or ≥2 rehospitalizations for respiratory illness.^34^ The secondary outcome was the composite outcome of death prior to 26 months corrected age or moderate-to-severe NDI at 22–26 months corrected age defined as any one of the following: BSID-III cognitive composite score <85 or ASQ score >2 SD below the mean on any domain,^32^ GMFCS score ≥2, moderate or severe CP, blindness, and/or severe hearing impairment that cannot be corrected by amplification.^30^ Other secondary outcomes included mortality, the components of the pulmonary and neurodevelopmental composite outcomes, parental report of chronic wheezing or cough, doctor/ER visits for respiratory illness, and respiratory medication use during the first 22–26 months corrected age.

### Statistical analysis

For the efficacy analysis, we compared the composite outcome death or serious respiratory morbidity at 22–26 months corrected age and other outcomes among all randomized participants according to the principle of intention-to-treat and in the subgroups of *Ureaplasma*-positive and negative participants. To account for possible correlation between outcomes in twins, we used generalized estimating equations^35^ and multiple outputation to calculate *p* values and confidence intervals.^36^ When observed counts were small, we used exact methods without accounting for twinning to calculate *p* values.

We did not collect a 22–26 month lung assessment for 15 of the children. To retain these children in the analyses described above, we multiply imputed the outcomes based on information gathered in the 6- and 12-month follow-up surveys and on respiratory variables observed prior to discharge. Binary outcomes were imputed based on their estimated probability. Twenty-five data sets with imputations were created and the final results were combined using Rubins approach.^37^

Treatment groups were compared with respect to the median value of several quantitative outcomes, including PMA at discharge to home and when supplemental oxygen was discontinued. To include children who died during the NICU hospitalization in the analysis appropriately, we used a rank-based (Wilcoxon test) analysis and gave these children the worst ranks.

In post hoc analyses, we explored the impact of upper and lower respiratory tract *Ureaplasma* colonization on the follow-up primary and major secondary outcomes.

All analyses were performed using SAS 9.4.

## Results

### Characteristics of the study population

**Fig. 1:**
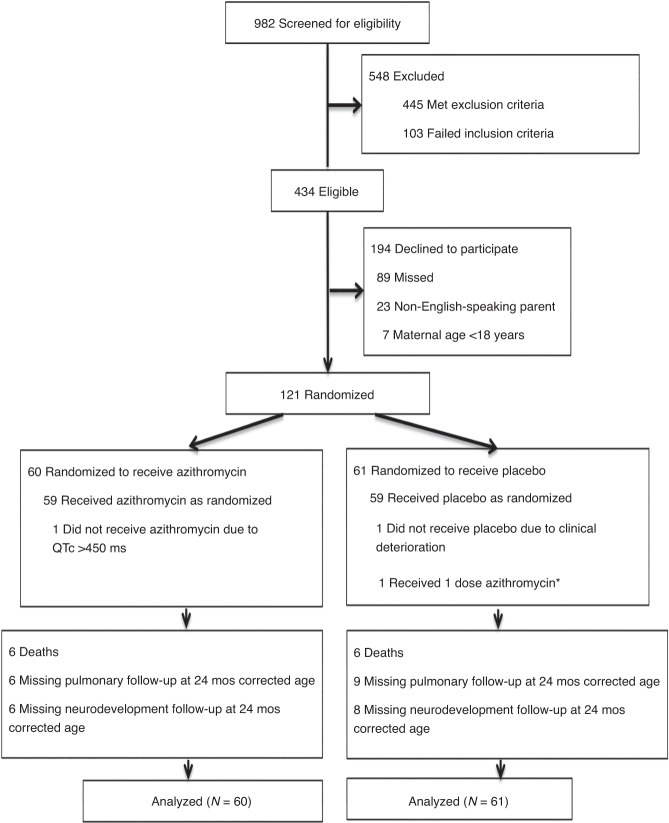
Consolidated standards of reporting trial diagram of the azithromycin in preterm trial. One participant in each treatment arm did not receive assigned treatment. Asterisk (*): one participant assigned to the placebo group, received one dose of azithromycin due to investigational pharmacy error but received placebo for other 2 doses. All participants were included in intent-to-treat analysis.

Of the 121 ELGAN infants randomized, 60 were assigned to AZM and 61 to placebo; 119 (98%) received at least one dose of the assigned treatment (Fig. 1).^21^ One placebo-assigned infant who was nasopharyngeal *Ureaplasma* positive received a single dose of AZM due to pharmacy error. The baseline characteristics of the study cohort and when stratified by *Ureaplasma* colonization status were similar between treatment arms, except for an imbalance in race distribution that occurred with 40% non-white in the AZM vs 75% in the placebo group (Table 1). Forty-four of the 121 (36%) participants were *Ureaplasma* positive at one or more timepoints with 19 randomized to AZM and 25 to placebo (Table 1).

**Table 1:** Characteristics of the study infants for the total cohort and stratified by *Ureaplasma* status.

	No. (%) of participants					
	Total cohort (*N* = 121)		*Ureaplasma* positive (*N* = 44)		*Ureaplasma* negative (*N* = 77)	
	AZM (*N* = 60)	Placebo (*N* = 61)	AZM (*N* = 19)	Placebo (*N* = 25)	AZM (*N* = 41)	Placebo (*N* = 36)
Baseline characteristics						
Gestational age, weeks, mean (SD)	26.2 (1.4)	26.2 (1.4)	25.8 (1.1)	25.8 (1.4)	26.4 (1.5)	26.5 (1.4)
24–26 weeks, *N* (%)	40 (67%)	43 (70%)	16 (84%)	20 (80%)	24 (59%)	23 (64%)
27–28 weeks, *N* (%)	20 (33%)	18 (30%)	3 (16%)	5 (20%)	17 (41%)	13 (36%)
Birth weight, g, mean (SD)	895 (215)	903 (245)	897 (195)	851 (282)	895 (226)	939 (213)
Sex, male, *n* (%)	26 (43%)	32 (52%)	11 (58%)	10 (40%)	15 (37%)	22 (61%)
Race, *N* (%)						
White	36 (60%)	15 (25%)	13 (68%)	5 (20%)	23 (56%)	10 (28%)
African-American	21 (35%)	43 (70%)	6 (32%)	19 (76%)	15 (37%)	24 (67%)
Asian	0 (0%)	1 (2%)	0 (0%)	1 (4%)	0 (0%)	0 (0%)
Multiple/biracial	3 (5%)	2 (3%)	0 (0%)	0 (0%)	3 (7%)	2 (6%)
Hispanic ethnicity, *N* (%)	2 (3%)	0 (0%)	0 (0%)	0 (0%)	2 (5%)	0 (0%)
Family history asthma, *N* (%)	35/52 (67%)	35/54 (65%)	10/15 (67%)	14/21 (67%)	25/37 (68%)	21/33 (64%)
Family history allergy, *N* (%)	33/52 (63%)	31/54 (57%)	11/15 (73%)	14/21 (67%)	22/37(59%)	17/33 (52%)
24–26 months corrected age characteristics of NICU survivors						
Corrected age at follow-up, month, mean (SD)	23.7 (2.1) (*n* = 48)	23.4 (1.7) (*n* = 46)	23.7 (2.1) (*n* = 15)	23.3 (1.9) (*n* = 19)	23.7 (2.1) (*n* = 33)	23.5 (1.5) (*n* = 27)
Caretaker marital status, married, *N* (%)	21/45 (47%)	16/47 (34%)	7/14 (50%)	5/19 (26%)	14/31 (45%)	11/28 (39%)
Insurance type, *N* (%)						
Medical Assistance	29 (60%)	35 (74%)	8 (53%)	16 (84%)	21 (64%)	19 (68%)
Private ± Medical Assistance	16 (33%)	9 (19%)	4 (27%)	2 (11%)	12 (36%)	7 (25%)
None	0 (0%)	2 (4%)	0 (0%)	0 (0%)	0 (0%)	2 (7%)
Unknown	3 (6%)	1 (2%)	3 (20%)	1 (5%)	0 (0%)	0 0%)
Caretaker level of education ≤high school, *N* (%)	13/48 (27%)	21/47 (45%)	6/15 (40%)	10/19 (53%)	7/33 (21%)	11/28 (39%)
Environmental smoke exposure, *N* (%)	16/56 (29%)	18/55 (33%)	6/16 (38%)	5/21 (24%)	10/40 (25%)	13/34 (38%)
Daycare attendance, *N* (%)	8/56 (14%)	11/55 (20%)	4/16 (25%)	6/21 (29%)	4/40 (10%)	5/34 (15%)

Eleven participants died prior to discharge from the NICU (AZM, *N* = 5; placebo, *N* = 6) and 1 AZM-assigned participant died after discharge and prior to 22–26-month corrected age. At least one post-discharge questionnaire was completed for 105/109 survivors (96%). At 22–26 months corrected age, 94 (86%) completed pulmonary assessments and 95 (87%) completed neurodevelopmental assessments (Fig. 1). Socioeconomic factors such as frequency of medical assistance insurance and maternal education and environmental factors such as daycare attendance and tobacco smoke exposure were similar between treatment arms. Family history of asthma and atopy was common for participants in both treatment arms.

### Primary pulmonary outcome

The primary composite pulmonary outcome of death prior to 22–26 months corrected age or serious respiratory morbidity was similar in the AZM (34.8%) compared to the placebo (30.4%) group (risk difference 4%, 95% confidence interval −14 to 21%, *p* = 0.67; Table 2) and in analyses stratified by race (Supplemental Table 1). The distribution of the components of the serious respiratory morbidity outcome was similar between the treatment groups (Supplemental Fig. 1). There were no differences in PMA at the time of discharge to home or when supplemental oxygen was discontinued. Among those positive for *Ureaplasma*, AZM-assigned participants had similar mortality (16% both groups) but significantly higher incidence of serious respiratory morbidity than placebo-assigned participants (45 vs 15%) (*p* = 0.036; Table 2). There was no difference in incidence of serious respiratory morbidity in *Ureaplasma*-negative infants. Parental report of chronic cough or wheezing was similar in both the treatment groups. Use of one or more respiratory medication during any interval in the first 2 years was high in both the groups (AZM, 65%; placebo, 62%), with nebulized albuterol as the most frequent medication used in both the groups. There were no appreciable differences in percentage of children who were hospitalized one or more times for respiratory illness between treatment arms and when stratified by *Ureaplasma* status.

**Table 2:** Pulmonary outcomes at 22–26 months corrected age of total cohort and stratified by *Ureaplasma* respiratory colonization status.

Outcome	No. (%) of participants								
	Total cohort (*N* = 121)			*Ureaplasma* positive (*N* = 44)			*Ureaplasma* negative (*N* = 77)		
	AZM (*N* = 60)	Placebo (*N* = 61)	*p* value*	AZM (*N* = 19)	Placebo (*N* = 25)	*p* value*	AZM (*N* = 41)	Placebo (*N* = 36)	*p* value*
PMA at discharge to home, median (IQR)^a^	39.2 (37.1, 44.4)	38.9 (37.0, 42.7)	0.74	40.4 (37.1, 43.3)	38.7 (36.9, 42.7)	0.63	39.1 (37.1, 44.4)	38.9 (37.4, 42.3)	0.92
PMA when supplemental O_2_ discontinued, weeks, median (IQR)^a^	38.0 (33.1, 60.4)	36.3 (32.9, 55.0)	0.92	44.3 (34.9, 123.9)	38.2 (34.6, 50.9)	0.36	36.9 (33.0, 44.6)	35.7 (31.1, 56.4)	0.91
Death or serious respiratory morbidity, *N* (%)^b^	20.9 (35%)	18.6 (30%)	0.67	10.2 (54%)	17.2 (29%)	0.064	10.7 (26%)	11.4 (33%)	0.53
All-cause mortality before 26 months corrected age, *N* (%)	6 (10%)	6 (10%)	0.97	3 (16%)	4 (16%)	1.0	3 (7%)	2 (6%)	1.0
Mortality from respiratory cause before 26 months corrected age, *N* (%)	3 (5%)	2 (3%)	0.68	2 (11%)	2 (8%)	1.0	1 (2%)	0 (0%)	1.0
Serious respiratory morbidity, *N* (%)^b,c^	14.9/54 (28%)	12.6/55 (23%)	0.63	7.1/16 (45%)	3.2/21 (15%)	0.036	7.7/38 (20%)	9.4/34 (28%)	0.36
Parental report chronic wheezing or chronic cough, *N* (%)^b,c^	16.0 (30%)	12.8 (23%)	0.62	4.1 (26%)	1.2 (6%)	0.23	12.0 (31%)	11.6 (34%)	0.72
≥1 hospitalization in first 22–26 months corrected age, *N* (%)^b,c^	18.6 (34%)	14.1 (26%)	0.33	6.1 (38%)	2.4 (11%)	0.14	12.5 (33%)	11.7 (34%)	0.90
Respiratory medication use, *N* (%)^b,c^	35.3 (65%)	34.4 (62%)	0.96	11.6 (73%)	13.4 (64%)	0.63	23.7 (62%)	21.0 (62%)	0.99
Diuretic use, *N* (%)^b,c^	7.4 (14%)	8.4 (15%)	0.59	4.0 (25%)	3.0 (14%)	0.44	3.4 (9%)	5.4 (16%)	0.49
Albuterol nebulizer use, *N* (%)^b,c^	30.2 (56%)	32.5 (59%)	0.69	8.5 (53%)	12.8 (61%)	0.79	21.8 (57%)	19.6 (58%)	0.99
Inhaled corticosteroid use, *N* (%)^b,c^	18.6 (35%)	20.3 (37%)	0.58	4.4 (28%)	7.3 (35%)	0.71	14.2 (37%)	13.0 (38%)	0.79
Oral prednisone, *N* (%)^b,c^	8.6 (16%)	5.2 (9%)	0.37	4.2 (27%)	2.0 (10%)	0.36	4.4 (11%)	3.2 (9%)	0.89

*p values are based on GEE to account for twins, except for binary variables with expected cell counts <5, in which case they were based on Fisher’s Exact Test, and except for comparisons of medians, which are based on a Wilcoxon test using multiple outputation to account for twins.

^a^11 patients who died before discharge were given the worst value in calculating the medians.

^b^The numerators for these variables are not always intergers due to the fact that we used multiple imputation of these outcomes for patients who were missing information on the 22–26-month follow-up.

^c^Based on survivors. *N* equals 54 for AZM and 55 for placebo patients. For *Ureaplasma* positives, *N* = 16 for AZM and *N* = 21 for placebo patients. For *Ureaplasma* negatives, *N* = 38 for AZM and *N* = 34 for placebo patients.

### Neurodevelopmental outcomes

NDI classification was successfully assigned for 93/109 (85%) survivors (BSID-III, *N* = 43; ASQ3, *N* = 44; and medical record review, *N* = 6; Table 3). The observed frequency of the composite outcome of death or moderate-to-severe NDI was 47% for the AZM treatment and 33% for the placebo arms for the entire cohort with risk difference 16% after adjusting for twins (95% confidence interval −2 to 34%, *p* = 0.11). There were no significant differences in composite outcome of death or moderate–severe NDI when stratified by *Ureaplasma* respiratory colonization status (Table 3) or race (Supplemental Table 1). There were no significant differences between treatment arms for the individual components of NDI, including the frequency of BSID-III composite cognitive score <85 or ASQ3 >2 SD below the mean in any domain and moderate–severe CP or GMFCS score ≥2. In addition, there was no difference between groups for BSID-III cognitive composite score using cutoff <70. Of note, infants who had any grade IVH had a significantly increased risk for moderate-to-severe NDI and cognitive impairment than infants without IVH after controlling for treatment in logistic regression analysis (*p* = 0.024) (Supplemental Fig. 2). No surviving participant was blind or had disabling hearing impairment.

**Table 3:** Neurodevelopmental outcomes at 22–26 months corrected age of total cohort and stratified by *Ureaplasma* respiratory colonization status.

Outcome	No. (%) of participants								
	Total cohort (*N* = 121)			*Ureaplasma* positive (*N* = 44)			*Ureaplasma* negative (*N* = 77)		
	AZM (*N* = 60)	Placebo (*N* = 61)	*p* value*	AZM (*N* = 19)	Placebo (*N* = 25)	*p* value*	AZM (*N* = 41)	Placebo (*N* = 36)	*p* value*
Death or moderate-to-severe NDI, *N* (%)^a^	25/53 (47%)	17/52 (33%)	0.11	7/18 (39%)	9/22 (41%)	0.99	18/35 (51%)	8/30 (27%)	0.077
Neurodevelopmental assessment tool, *N* (%)^b^									
BSID-III	18 (38%)	25 (54%)	0.31	7 (47%)	8 (44%)	0.63	11 (34%)	17 (61%)	0.15
ASQ3	26 (55%)	18 (39%)		8 (53%)	8 (44%)		18 (56%)	10 (36%)	
Medical record review	3 (6%)	3 (6%)		0 (0%)	2 (11%)		3 (9%)	1 (4%)	
BSID-III cognitive composite score <85 or ASQ3 <2 SD any domain, *N* (%)^b^	18/47 (38%)	11/46 (24%)	0.14	4/15 (27%)	5/18 (28%)	1.0	14/32 (44%)	6/28 (21%)	0.15
BSID-III cognitive score <85^c^	7/17 (41%)	7/25 (28%)	0.39	4/6 (67%)	6/8 (75%)	1.0	5/11 (45%)	5/17 (29%)	0.75
BSID cognitive score <70^c^	4/17 (24%)	3/25 (12%)	0.66	1/6 (17%)	0/8 (0%)	0.43	3/11 (27%)	3/17 (18%)	0.65
Moderate-to-severe CP with GMFCS level ≥2, *N* (%)	7/47 (15%)	2/47 (4%)	0.16	3/15 (20%)	1/19 (5%)	0.30	4/32 (13%)	1/28 (4%)	0.36
Blindness, *N* (%)^b^	0/48 (0%)	0/47 (0%)	N/A	0/15 (0%)	0/19 (0%)	N/A	0/33 (0%)	0/28 (0%)	N/A
Deafness, *N* (%)^b^	0/47 (0%)	0/47 (2%)	N/A	0/15 (0%)	0/19 (0%)	N/A	0/31 (0%)	0/28 (4%)	N/A

*N/A* not applicable.

*p values are based on GEE to account for twins, except for binary variables with expected cell counts <5, in which case they were based on Fisher’s Exact Test.

^a^Based on those who had a 22–26-month corrected age neurologic assessment (48 AZIP, 47 placebo) or who died.

^b^Based on 95 patients who had a 22–26-month corrected age neurologic assessment (48 AZIP, 47 placebo) minus those patients whose assessment was inconclusive due to missing some items.

^c^Based on 42 patients who received a BSID-III assessment (17 AZM, 25 placebo).

### Post hoc analyses

We had previously observed that *Ureaplasma*-free survival, overall survival, and physiologic BPD-free survival were lower and durations of hospitalization, mechanical ventilation and supplemental oxygen, and postnatal steroid exposure were higher in intubated patients with lower respiratory tract *Ureaplasma* colonization compared to intubated infants without lower tract involvement or non-intubated infants.^21^ At follow-up, differences in unfavorable pulmonary outcomes persisted in children who had lower respiratory tract *Ureaplasma* colonization. Tracheal aspirate *Ureaplasma*-positive infants were discharged to home and supplemental oxygen was discontinued at later PMA and they experienced a higher frequency of the composite outcome death or serious respiratory morbidity at 22–26 months corrected age than tracheal aspirate *Ureaplasma*-negative infants and non-intubated infants (Table 4). There were no differences among the three groups for death or moderate-to-severe NDI. To assess the long-term effect of neonatal lower respiratory tract *Ureaplasma* colonization in the absence of treatment, we compared outcomes of placebo-treated tracheal aspirate *Ureaplasma*-positive and *Ureaplasma*-negative infants (Supplemental Table 2). Placebo-treated tracheal aspirate *Ureaplasma*-positive infants were discharged to home at a later PMA. Although the differences were not statistically significant, all-cause mortality (36 vs 9%, *p* = 0.07) and mortality from respiratory causes (18 vs 0%, *p* = 0.098) was greater in the *Ureaplasma*-positive group than for infants assigned to placebo. In patients with lower respiratory tract *Ureaplasma* colonization, death or serious respiratory morbidity was 60% (6/10) in AZM-treated vs 56% (6/11) in placebo-treated infants (*p* = 0.61; Table 5).

**Table 4:** Pulmonary and neurodevelopmental outcomes at 22–26 months corrected age of participants on non-invasive respiratory support, invasive ventilation with tracheal aspirate *Ureaplasma*-negative specimens, and invasive ventilation with tracheal aspirate *Ureaplasma*-positive specimens.

Outcome	No. (%) of participants			*p* value*
	Never intubated (no TA specimen) (*N* = 47)	TA *Ureaplasma* negative (*N* = 52)	TA *Ureaplasma* positive (*N* = 21)	
PMA at discharge to home, weeks, median (IQR)^a^	37.7 (36.4, 40.9)	41.6 (38.9, 47.3)	45.6 (39.9, ??)	<0.0001
PMA when supplemental oxygen discontinued, weeks, median (IQR)^a^	33.9 (29.6, 37.6)	40.8 (34.6, 72.2)	50.9 (38.4, ??)	<0.0001
Death or serious respiratory morbidity, *N* (%)^b^	9.7 (21)	17.4 (34%)	12.1 (58%)	0.028
All-cause mortality before 26 months corrected age, *N* (%)	0/47 (0%)	6/52 (12%)	6/21 (29%)	0.0006
Mortality from respiratory cause before 26 months corrected age, *N* (%)	0/47 (0%)	2/52 (4%)	3/21 (14%)	0.028
Serious respiratory morbidity, *N* (%)^b,c^	9.7 (21%)	11.4 (25%)	6.1 (41%)	0.20
Parental report chronic wheezing or chronic cough, *N* (%)^b,c^	10.6 (22%)	15.6 (34%)	2.2 (14%)	0.32
≥1 hospitalization in the first 22–26 months corrected age, *N* (%)^b,c^	14.8 (31%)	13.4 (31%)	3.2 (21%)	0.66
Respiratory medication use, *N* (%)^b,c^	29.9 (64%)	28.0 (61%)	11.4 (76%)	0.58
Death or moderate-to-severe NDI, *N* (%)^d^	14/42 (33%)	19/44 (43%)	9/19 (47%)	0.60
BSID-III cognitive composite score <85 or ASQ3 <2 SD any domain, *N* (%)^d^	14/42 (33%)	12/38 (32%)	3/13 (23%)	0.87
BSID-III cognitive score <85, *N* (%)^d^	6/22 (27%)	6/15 (40%)	2/5 (40%)	0.65
BSID-III cognitive score <70, *N* (%)^d^	3/22 (14%)	3/15 (20%)	1/5 (20%)	0.86
Moderate-to-severe CP with GMFCS level ≥2, *N* (%)^d^	2/42 (5%)	5/38 (13%)	2/14 (14%)	0.31
Blindness, *N* (%)^d^	0/43 (0%)	0/38 (0%)	0/14 (0%)	N/A
Deafness, *N* (%)^d^	0/41 (0%)	0/38 (0%)	0/14 (0%)	N/A

Since deaths represented >25% in the TA *Ureaplasma*-positive group, it was not possible to determine the third quartile of the distribution for outcomes in this group (represented by ??).

*N/A* not applicable.

*p values are based on GEE to account for twins, except for binary variables with expected cell counts <5, in which case they were based on Fisher’s Exact Test, and except for comparing medians, which was based on Kruskal–Wallis test using multiple outputation to account for twins.

^a^11 patients who died before discharge were given the worst value in calculating the medians.

^b^The numerators for these variables are not always integers due to the fact that we used multiple imputation of these outcomes for patients who were missing information on the 22–26-month corrected age follow-up.

^c^Based on survivors (*N* = 47, 46, and 15 for the three columns, respectively).

^d^Based on those who had a 22–26-month corrected age neurodevelopment assessment.

**Table 5:** Pulmonary and neurodevelopmental outcomes at 22–26 months corrected age among tracheal aspirate *Ureaplasma*-positive participants by treatment assignment.

Outcome	No. (%) of participants		*p* value*
	Azithromycin (*N* = 10)	Placebo (*N* = 11)	
PMA at discharge to home, weeks, median (IQR)^a^	39.4 (38.1, 84.1)	52.4 (43.9, ?)	0.094
PMA when supplemental oxygen discontinued, weeks, median (IQR)^a^	53.3 (34.9, 123.9)	50.9 (41.6, ?)	0.45
Death or serious respiratory morbidity, *N* (%)^b^	6.0 (60%)	6.1 (56%)	0.61
All-cause mortality before 26 months corrected age, *N* (%)	2/10 (20%)	4/11 (36%)	0.64
Mortality from respiratory cause before 26 months corrected age, *N* (%)	1/10 (10%)	2/11 (18%)	1.0
Serious respiratory morbidity, *N* (%)^b,c^	4.0 (50%)	2.1 (30%)	0.30
Parental report chronic wheezing or chronic cough, *N* (%)^b,c^	2.0 (25%)	0.2 (2%)	0.56
Respiratory medication use, *N* (%)^b,c^	6.0 (75%)	5.4 (77%)	1.0
Death or moderate-to-severe NDI, *N* (%)^d^	4/10 (40%)	5/9 (56%)	0.66
BSID-III cognitive composite score <85 or ASQ3 <2 SD any domain, *N* (%)^d^	2/8 (25%)	1/5 (20%)	1.0
Moderate-to-severe CP with GMFCS level ≥2, *N* (%)^d^	2/8 (25%)	0/6 (0%)	0.47
Suspected blindness, *N* (%)^d^	0/8 (0%)	0/6 (0%)	N/A
Deafness or suspected deafness, *N* (%)^d^	0/8 (0%)	0/6 (0%)	N/A

Since deaths represented more than 25% in the TA *Ureaplasma*-positive group, it was not possible to determine the third quartile of the distribution of these outcomes in this group (represented by ?).

*N/A* not applicable.

*p values based on Wilcoxon or Fisher’s Exact Test.

^a^11 patients who died before discharge were given the worst value in calculating the medians.

^b^The numerators for these variables are not always integers due to the fact that we used multiple imputation of these outcomes for patients who were missing information on the 22–26-month corrected age follow-up.

^c^Based on survivors (*N* = 8 and *N* = 7 for the two columns, respectively).

^d^Based on those who had a 22–26-month corrected age neurodevelopment assessment.

## Discussion

We conducted 22–26-month corrected age follow-up for this Phase IIb RCT to assess the potential impact of AZM therapy on long-term pulmonary and neurodevelopmental outcomes. We have previously published the primary efficacy outcome analysis showing that intravenous AZM 20 mg/kg for 3 days effectively eradicates *Ureaplasma* respiratory colonization.^21^ In the follow-up study, we did not find strong evidence for an impact of neonatal exposure to AZM on later pulmonary and neurodevelopment outcomes. Based on the 95% confidence interval (−14 to 21%), our data do not rule out either a moderately reduced or moderately increased risk of death or serious respiratory morbidity at 22–26 months corrected age in those receiving AZM relative to placebo. Similarly, the 95% confidence interval for difference in death or NDI between treatment groups is broad (−2 to 34%). Importantly, we observed that infants who had lower respiratory tract *Ureaplasma* colonization in the neonatal period had persistent adverse pulmonary outcomes, including greater risk for the composite outcome death or serious respiratory morbidity, suggesting that these infants should be targeted in future trials.

Since the widely used BPD definitions are poor predictors of subsequent respiratory illness later in childhood, consensus is lacking among neonatologists on which clinically meaningful short- and long-term endpoints should be targeted in clinical trials of BPD prevention.^23,38–40^ In the current follow-up study, we chose the composite outcome of death or serious respiratory morbidity defined by Jensen et al.^34^ as the primary outcome. The components of the serious respiratory morbidity definition could be ascertained from the electronic medical records for the majority of subjects and they represent meaningful clinical outcomes^7^ and are factors that impact parents’ perceptions of quality of life for their children^41^ and inform healthcare providers.^5^ Jensen et al.^34^ determined that a revised BPD definition that categorized lung disease severity according to mode of respiratory support at 36 weeks PMA rather than supplemental oxygen best predicted serious respiratory morbidity in the first 2 years of life in preterm infants born <27 weeks gestation.

Questionnaires that rely on parental recall of respiratory symptoms, medication use, and physician visits have been commonly used in studies of pulmonary outcomes in preterm infants.^27,42,43^ The potential for parental recall bias of respiratory symptoms such as wheezing has been debated in previous studies.^44,45^ To reduce recall bias in the current study, we used the validated Tucson Children’s Respiratory Study questionnaires administered by trained personnel at 4 timepoints during the first 2 years of life. Approximately two-thirds of parents reported family history of asthma and/or atopy. Respiratory medication use prevalence was ~65% in surviving children in both treatment arms with nebulized albuterol the most frequently reported medication. Respiratory medication use was similar to rates reported in other ELGAN cohorts during the first few years of life.^27,34,43^ Whether these medications were prescribed for BPD or viral infection-related wheezing is not known.

*Ureaplasma* respiratory tract colonization has been proposed as an etiologic factor in reactive airway disease in young infants. Wheezing in infants and children <3 years of age has been associated with isolation of *Ureaplasma* from the upper respiratory tract.^46^ There was no significant difference in parental-reported chronic cough or wheezing in children who were *Ureaplasma* tracheal aspirate positive compared to those children who had been tracheal aspirate negative or never intubated. In post hoc analysis of placebo-treated intubated infants with and without lower tract *Ureaplasma* colonization, *Ureaplasma*-colonized infants were discharged to home at later PMA, suggesting a more complicated NICU course in colonized infants.

We examined the impact of AZM treatment in the first few days of life on later NDI, a treatment effect that has not been previously reported. The rate of death or moderate-to-severe NDI in the current study cohort is similar to rates reported in other neonatal cohorts.^30,34^ Although AZM has been proposed as a neuroprotective agent in neonatal animal models,^47^ there was no apparent benefit in neurodevelopmental measures by treatment group or when stratified by *Ureaplasma* status. Despite worse pulmonary morbidity, infants with lower respiratory tract *Ureaplasma* infection had similar rate of death or moderate–severe NDI as intubated infants without lower tract involvement or non-intubated infants. In multiple previous studies, BPD severity was a risk factor for adverse neurodevelopmental outcomes.^7,8^ However, in a recent single-center birth cohort, BSID-III cognitive scores were not associated with BPD severity.^48^

AZM has a low incidence of side effects in adults and children aged >2 years, primarily gastrointestinal symptoms.^49^ The safety of AZM in preterm infants has been assessed in a systematic review of 11 neonatal RCTs and observational studies.^17^ The majority of adverse events in the neonatal period were related to prematurity and were assessed as unlikely associated with AZM treatment. Although infantile hypertrophic pyloric stenosis has been associated with AZM exposure in the first few weeks of life,^50,51^ only 4 cases were identified in 473 infants included in the systematic review and there were no cases in the current trial. In addition, AZM is pro-arrhythmogenic with prior reports of occurrences of QT-interval prolongation and torsade’s de pointes in adults^52^ but has not been observed in neonates treated with therapeutic doses.

Long-term safety of neonatal AZM exposure has not been previously addressed. In our small sample of children who were *Ureaplasma* positive from upper and/or lower airways, there was significantly greater serious respiratory morbidity in the AZM-assigned group than in the placebo-assigned group. A larger trial will be needed to determine whether this finding is directly related to neonatal AZM exposure or is due to random variation. This observation underscores the importance of delaying adopting AZM therapy to prevent BPD into clinical practice until a Phase III RCT is completed. There is an ongoing AZM therapy for chronic lung disease of prematurity (AZTEC) Phase III RCT of a 10-day course of AZM (20 mg/kg × 3 days, followed by 10 mg/kg for a further 7 days) that will enroll 796 preterm infants <30 weeks gestation to determine the safety and efficacy of this regimen to improve survival without BPD.^53^

### Study limitations

Our sample size had good power to detect a treatment effect on the primary outcome of clearance of *Ureaplasma*, but we had insufficient power to exclude small-to-moderate differences between the groups for long-term pulmonary and neurodevelopmental outcomes. To indicate the range of group differences that could not be ruled out with our data, we presented 95% confidence intervals. Also, we recognize that relationships suggested early in follow-up—close in time to characterization with regard to *Ureaplasma* or AZM treatment—can be expected to attenuate as other exposures such as viral illnesses and differences in home environment influence pulmonary and neurological outcomes, further eroding statistical power. Attrition at the 22–26-month corrected age assessment timepoint may have introduced ascertainment bias since higher rates of NDI are significantly correlated with increased loss to follow-up in a systematic review of 43 follow-up studies of ELGAN infants.^54^

### Study implications summary

The results of this trial extend the observation that mortality and prolonged respiratory support are concentrated in ELGANs who have *Ureaplasma* in the lower respiratory tract and inform the design of a future Phase III trial targeting *Ureaplasma*-colonized infants. An adequately powered Phase III clinical trial limited to ELGAN infants with PCR-confirmed lower respiratory tract *Ureaplasma* would determine whether or not a 3-day course of AZM is of clinical benefit to reduce short- and long-term pulmonary morbidity.

## Supplementary information

Supplementary Information
