# Supplementary material for: Randomized trial of azithromycin to eradicate Ureaplasma respiratory colonization in preterm infants: 2-year outcomes
Source: Pediatr Res. 2021 Mar 3;91(1):178–87. doi: 10.1038/s41390-021-01437-2 (PMC8413397; doi:10.1038/s41390-021-01437-2)
Supplement: Supplementary file 1 — Supplementary Information [file 41390_2021_1437_MOESM1_ESM.pdf]

DATA SUPPLEMENT

Randomized Trial of Azithromycin to Eradicate *Ureaplasma* Respiratory Colonization in Preterm Infants: Two Year Outcomes

Rose M. Viscardi, MD[1*]; Michael L. Terrin, MD[2]; Laurence S. Magder, PhD[2]; Natalie L. Davis, MD[1]; Susan J. Dulkerian, MD[1]; Ken B. Waites, MD[3]; Marilee Allen, M.D.[4]; Ajoke Ajayi-Akintade, M.D.[5]; Namasivayam Ambalavanan, MD[3]; David A. Kaufman, MD[6]; Pamela Donohue, ScD[4], Deborah J. Tuttle, MD[7]; Jörn-Hendrik Weitkamp, MD[8]

[1]Departments of Pediatrics, and [2]Epidemiology and Preventive Medicine, University of Maryland, Baltimore School of Medicine, Baltimore, MD; [3]Departments of Pathology and Pediatrics, University of Alabama at Birmingham School of Medicine, Birmingham, AL; [4]Department of Pediatrics, Johns Hopkins University School of Medicine, Baltimore, MD; [5]Mount Washington Pediatric Hospital, Baltimore, MD; [6]Department of Pediatrics, University of Virginia School of Medicine, Charlottesville, VA; and [7]Department of Pediatrics, Christiana Care Health System, Newark, DE; [8]Department of Pediatrics, Vanderbilt University Medical Center, Nashville, TN.

[*]Corresponding Author: Rose M. Viscardi (email:rviscard@som.umaryland.edu), telephone: 410-328-6003), Professor of Pediatrics and Medicine, University of Maryland School of Medicine, 110 Paca Street, 8th floor, Baltimore, MD 21201, United States

LIST OF ELEMENTS IN THIS DATA SUPPLEMENT

1. Supplementary Table 1S. Primary and Secondary Outcomes, Stratified by Race
2. Supplementary Table 2S. Pulmonary and Neurodevelopmental Outcomes at 22-26 Months Corrected Age of Placebo-assigned Tracheal Aspirate *Ureaplasma*-negative and Tracheal Aspirate *Ureaplasma*-positive Participants.
3. Supplementary Figure S1. Serious Respiratory Morbidity in Participants Randomized to Azithromycin and Placebo
4. Supplementary Figure S2. Neurodevelopmental Impairment at 22-26 Months Corrected Age in Neonates Randomized to Azithromycin and Placebo with and without Intraventricular Hemorrhage

Supplemental Table S1. Pulmonary and Neurodevelopmental Outcomes at 22-26 Months Corrected Age Stratified by Race

	White (N=51)			Non-White (N=70)		
Outcome	Azithromycin (N=36)	Placebo (N=15)	P value[*]	Azithromycin (N=24)	Placebo (N=46)	P value[*]
PMA at discharge to home, wks, median (IQR)[a]	40.8 (38.3, 45.9)	39.7 (38.6, 41.7)	0.19	37.3 (36.4, 40.9)	38.4, (37.0, 42.9)	0.20
PMA when supplemental O2 discontinued, wks, median (IQR)[a]	40.8 (33.6, 67.1)	38.3 (35.0, 74.3)	0.77	34.9 (31.6, 42.4)	36.0 (31.6, 55.0)	0.40
Death or serious respiratory morbidity, N (%)[b]	12.6 (35%)	4.4 (29%)	0.80	8.3 (35%)	14.2 (31%)	0.72
All cause mortality before 26 months corrected age, N (%)	5/36 (14%)	2/15 (13%)	1.0	1/24 (4%)	4/46 (9%)	0.65
Mortality from respiratory cause before 26 months corrected age, N (%)	2/36 (6%)	1/15 (7%)	1.0	1/24 (4%)	1/46 (2%)	1.0
Serious respiratory morbidity, N (%)[c]	7.6 (25%)	2.4 (18%)	0.83	7.3 (32%)	10.2 (24%)	0.46
Death or moderate to severe NDI, N (%)[d]	14/32 (44%)	5/11 (45%)	0.40	11/22 (50%)	12/42 (29%)	0.13
BSIDIII cognitive composite score<85 or ASQ3 <2SD any domain, N (%)[d]	8/27 (30%)	3/9 (33%)	1.0	10/20 (50%)	8/37 (22%)	0.084
BSIDIII cognitive score <85[d]	4/10 (40%)	2/6 (33%)	1.0	3/7 (43%)	5/19 (26%)	0.64
BSIDIII cognitive score <70[d]	1/10 (10%)	1/6 (17%)	1.0	3/7 (43%)	2/19 (11%)	0.10
Moderate to severe CP with GMFCS level ≥2, N (%)[d]	4/27 (15%)	0/9 (0%)	0.55	3/20 (15%)	2/38 (5%)	0.33
Blindness, N (%)[d]	0/27 (0%)	0/9 (0%)	NA	0/21 (0%)	0/38 (0%)	N/A
Deafness, N (%)[d]	0/27 (0%)	0/9 (0%)	1.0	0/20 (5%)	0/38 (0%)	N/A

[*]P-values are based on GEE to account for twins, except for binary variables with expected cell counts less than 5, in which case they were based on Fisher's Exact Test.
[a] 11 patients who died before discharge were given the worst value in calculating the medians,
[b] The numerators for these variables are not always integers due to the fact that we used multiple imputation of these outcomes for 11 patients who were missing information on the 24-month follow-up.
[c] Based on survivors.
[d]Based on those who had a 24-month neurodevelopment assessment.

Table 2S. Pulmonary and neurodevelopmental outcomes at 22-26 months corrected age of placebo-assigned tracheal aspirate *Ureaplasma*-negative and tracheal aspirate *Ureaplasma*-positive participants.

	Number of Participants (%)		
Outcome	TA *Ureaplasma* Negative (N=23)	TA *Ureaplasma* Positive (N=11)	P value*
PMA at discharge to home, median (IQR)[a]	41.1 (38.7, 49.6)	52.4 (43.9, ??)	0.014
PMA when supplemental O2 discontinued, wks, median (IQR)[a]	42.7 (35.0, 76.1)	50.9 (41.6, ??)	0.15
Death or serious respiratory morbidity, N (%)[b]	9.1 (39%)	6.1 (56%)	0.38
All cause mortality before 26 months corrected age, N (%)	2/23 (9%)	4/11 (36%)	0.070
Mortality from respiratory cause before 26 months corrected age, N (%)	0/23 (0%)	2/11 (18%)	0.098
Serious respiratory morbidity, N (%)[b,c]	7.1 (34%)	2.1 (30%)	0.87
Parental report chronic wheezing or chronic cough, N (%) [b,c]	7.2 (34%)	0.2 (2%)	0.26
≥1 hospitalization in the first 22-26 months corrected age, N (%) [b,c]	7.4 (35%)	0.2 (2%)	0.25
Respiratory Medication Use, N (%)[b,c]	13.3 (63%)	5.4 (77%)	0.54
Death or moderate to severe NDI, N (%)[d]	7/18 (39%)	5/9 (56%)	0.45
BSIDIII cognitive composite score<85 or ASQ3 < 2SD any domain, N (%)[d]	5/16 (31%)	1/5 (20%)	1.0
BSIDIII cognitive score <85[d]	4/8 (50%)	1/ 2 (50%)	1.0
BSIDIII cognitive score <70[d]	2/8 (25%)	0/2 (0%)	1.0
moderate to severe CP with GMFCS level ≥2, N (%)[d]	1/16 (6%)	0/6 (0%)	1.0
Blindness, N (%) [d]	0/16 (0%)	0/6 (0%)	N/A
Deafness, N (%)[d]	0/16 (0%)	0/6 (0%)	N/A

*P-values are based on GEE to account for twins, except for binary variables with expected cell counts less than 5, in which case they were based on Fisher's Exact Test, and except for comparing medians which was based on the Wilcoxon test

[a]6 patients who died before discharge were given the worst value in calculating the medians. For that reason, the 3rd quartile could not be calculated for some subgroups.

[b] The numerators for these variables are not always integers due to the fact that we used multiple imputation of these outcomes for patients who were missing information on the 22-26-month follow-up.

[c]Based on survivors (N=21 TA *Ureaplasma*-negative; N=7 TA *Ureaplasma*-positive).

[d]Based on those who had a 24-month neurodevelopment assessment.

Supplemental Figure 1S

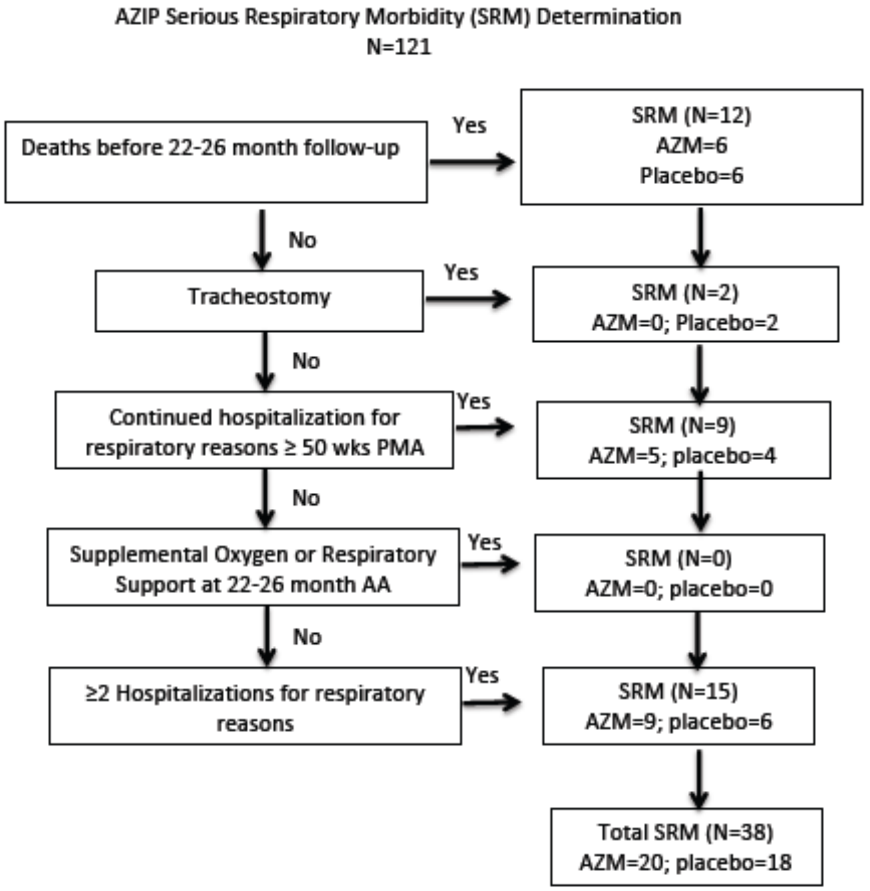

Supplemental Figure S1. Flow diagram of serious respiratory morbidity (SRM) determination in infants randomized to azithromycin (AZM) or placebo. SRM was defined as the occurrence of one or more of the following: tracheostomy; continued hospitalization for respiratory reasons at or beyond 50 weeks PMA, use of supplemental oxygen or respiratory support at 22-26 months adjusted age, or ≥2 rehospitalizations for respiratory illness (Jensen, E. A., et al. The Diagnosis of Bronchopulmonary Dysplasia in Very Preterm Infants. An Evidence-based Approach. *Am. J. Respir. Crit. Care Med.* **200**:751-759 (2019).

Supplemental Figure S2.

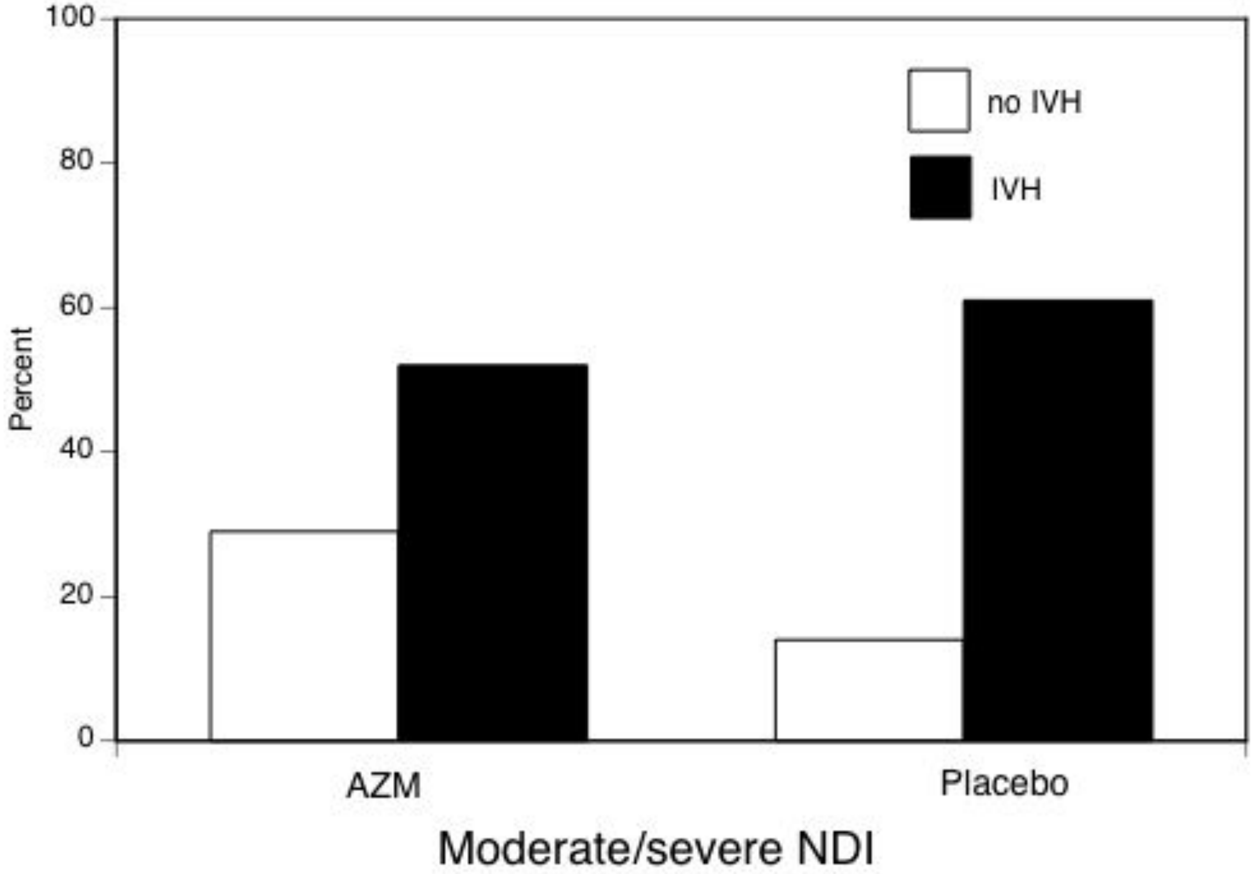

Supplemental Figure S2. Comparison of frequency of moderate-to-severe neurodevelopmental impairment in infants at 22-26 months AA randomized to azithromycin (AZM) or placebo with or without any grade IVH. Infants with any grade IVH had a significantly increased risk of moderate-to-severe NDI after controlling for treatment based on GEE logistic regression (p=0.024)
